# Supplementary material for: Separation and Loss of Centrioles From Primordidal Germ Cells To Mature Oocytes In The Mouse
Source: Sci Rep. 2018 Aug 24;8:12791. doi: 10.1038/s41598-018-31222-x (PMC6109097; doi:10.1038/s41598-018-31222-x)
Supplement: Supplementary file 1 — Supplementary Information [file 41598_2018_31222_MOESM1_ESM.pdf]

Supplemental Information.

SEPARATION AND LOSS OF CENTRIOLES FROM

PRIMORDIDAL GERM CELLS TO MATURE OOCYTES IN THE MOUSE

by

Calvin Simerly[1], Marion Manil-Segalen[3], Carlos Castro[1], Carrie Hartnett[1], Dong Kong[2],

Marie-Helene Verlhac[3], Jadranka Loncarek[2], and Gerald Schatten[1,*]

[1]Departments of Cell Biology; Obstetrics, Gynecology and Reproductive Sciences; and Bioengineering
University of Pittsburgh School of Medicine, Pittsburgh, PA 15213
[2]Laboratory of Protein Dynamics and Signaling, National Institutes of Health/Center for Cancer Research/National Cancer Institute-Frederick, Frederick, MD 21702
[3] Center for Interdisciplinary Research in Biology (CIRB) College de France, CNRS, INSERM, PSL Research University, Equipe labellisée FRM, Paris, France.verlhac@college-de-france.fr

[*]Corresponding author; email: gschatten@pdc.magee.edu

Supplemental Figures.

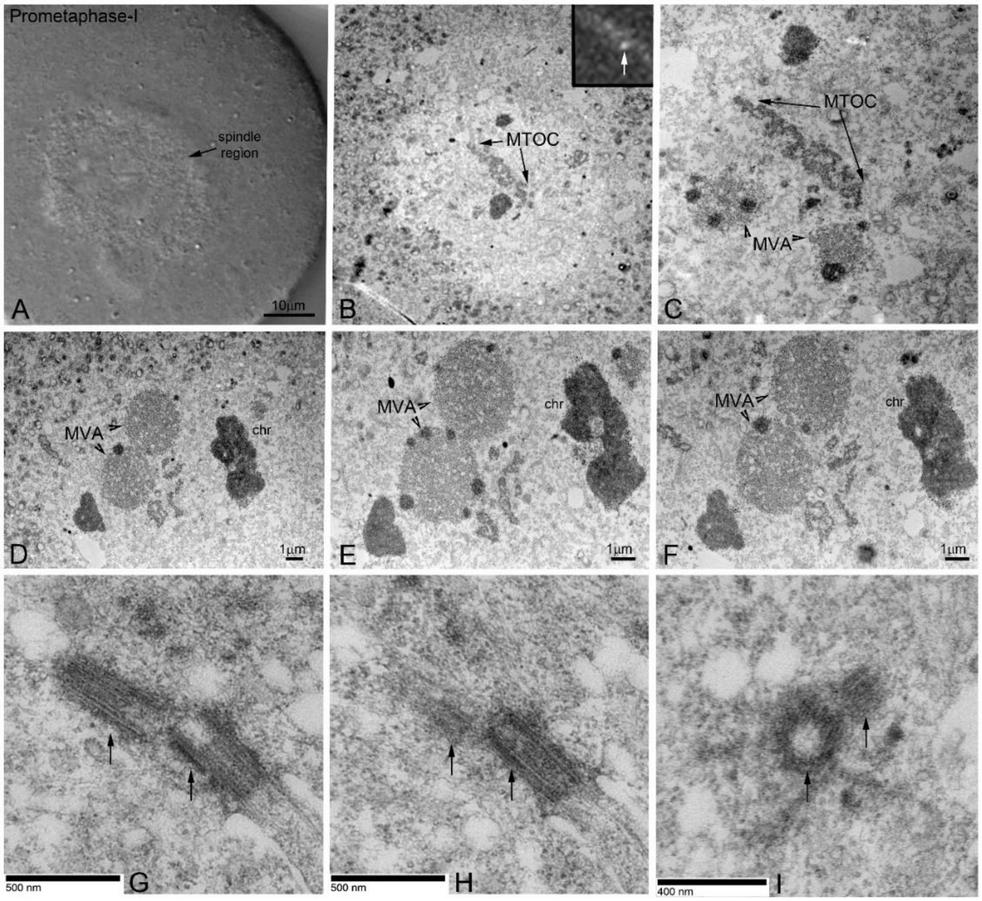

Supplemental Figure S1. Correlative light and electron microscopy (CLEM) analysis of GFP CETN2-expressing prometaphase-I oocytes for centriole detection. A: DIC image of the prometaphase-I GFP CETN2 expressing oocyte showing assembling spindle region (arrow) analyzed for centrioles. **B-F**: representative 200-nm EM sections through the prometaphase-I oocyte showing linear MTOCs (arrows), multivesicular aggregates (MVAs; arrowheads), vacuoles and other dense osmiophilic foci but no 9-triplet microtubule structures indicative of canonical centrioles in the spindle region. **B**, inset: detection of GFP CETN2 (arrow) in live oocyte at the MTOC ribbon prior to fixation and processing for electron microscopy. In **D-F**, MVA structures devoid of membranes (arrowheads) are shown adjacent to a condensing bivalent (chr). No 9-triplet microtubule structures are visible despite small spherical structures within the MVAs. **G-I**: unlike the failure to see centrioles in assembling spindles, 200-nm EM sections through cumulus cells attached to the prometaphase-I oocyte shows a pair of classical centrioles in longitudinal (G and H: arrows) and cross-section (I: arrows) configurations. Bars, as marked.

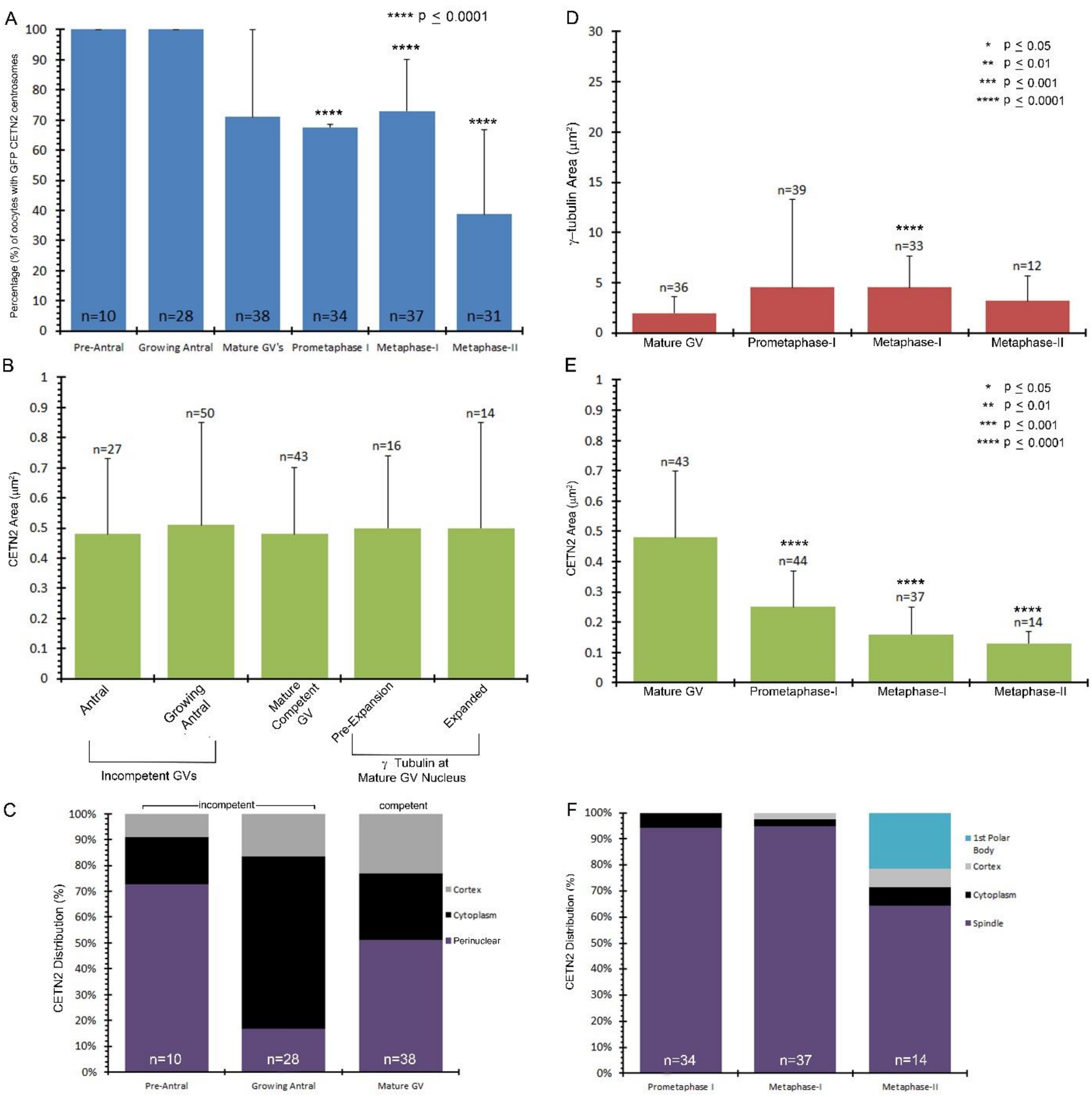

Supplemental Figure S2. GFP CETN2 expressing centrioles in mature, metaphase-II arrested oocytes and first polar bodies. (**A-K**): GFP CETN2 expressing centriole pairs found in three localizations, including a single spindle pole edge (A: green, long arrow; B: details) embedded in γ–tubulin (A: red), within the cytoplasm lacking γ–tubulin (A: green, short arrow; red, γ–tubulin; C: details), and the first polar body (1st Pb; A: green, arrowhead) without γ–tubulin (A: red; D: details). Only the spindle associated GFP CETN2 centrioles (F: green, long arrow) are with assembled microtubules (F: red, G: details). Small cytoplasmic microtubule ringlets (F: red, double arrowheads) lie near the cytoplasmic GFP CETN2 centriole pair (F: green, short arrow; H: details). No microtubules nucleate from the 1st polar body GFP CETN2-

expressing centrioles (F: green, arrowhead; red, microtubules; blue, DNA). E, J: rotational views (axis, lower right in panels) showing GFP CETN2 (green), γ–tubulin (red) and either DNA (E: blue) or microtubules (J: blue). (**L-T**): GFP CETN2 expressing centriole pairs at a single spindle pole (L: green, arrow) within a large focus of γ–tubulin (L: red; M: details). The elicited 1st polar body has a GFP CETN2 expressing centriole pair (L: green, arrowhead; N: details) with faint γ–tubulin foci (L: red; best observed in O, S panels; red). The GFP CETN2 expressing centriole pair at the spindle pole (P: green, long arrow) is within assembled spindle microtubules (P: red; Q: details) while the 1st polar body GFP CETN2 expressing centrioles do not intersect with the disorganized midbody microtubules (P: 1st PB, arrowhead; red, microtubules; R: details). Cytasters not expressing GFP CETN2 are identified in the cytoplasm (P: red, short arrows). O, S: rotational views (axis, lower right in panels) of oocytes for GFP CETN2 expressing centriole pairs (green), γ–tubulin (red) and either DNA (O: blue) or microtubules (S: blue). Images are direct expressing GFP CETN2 oocytes counterstained for γ–tubulin (red), DNA (blue) and microtubules (cy5, color coded either red or blue). K, T: Differential interference contrast (DIC) images with * denoting spindle regions and first polar bodies (1st Pb). Cyto: cytoplasm. Bars, μm.

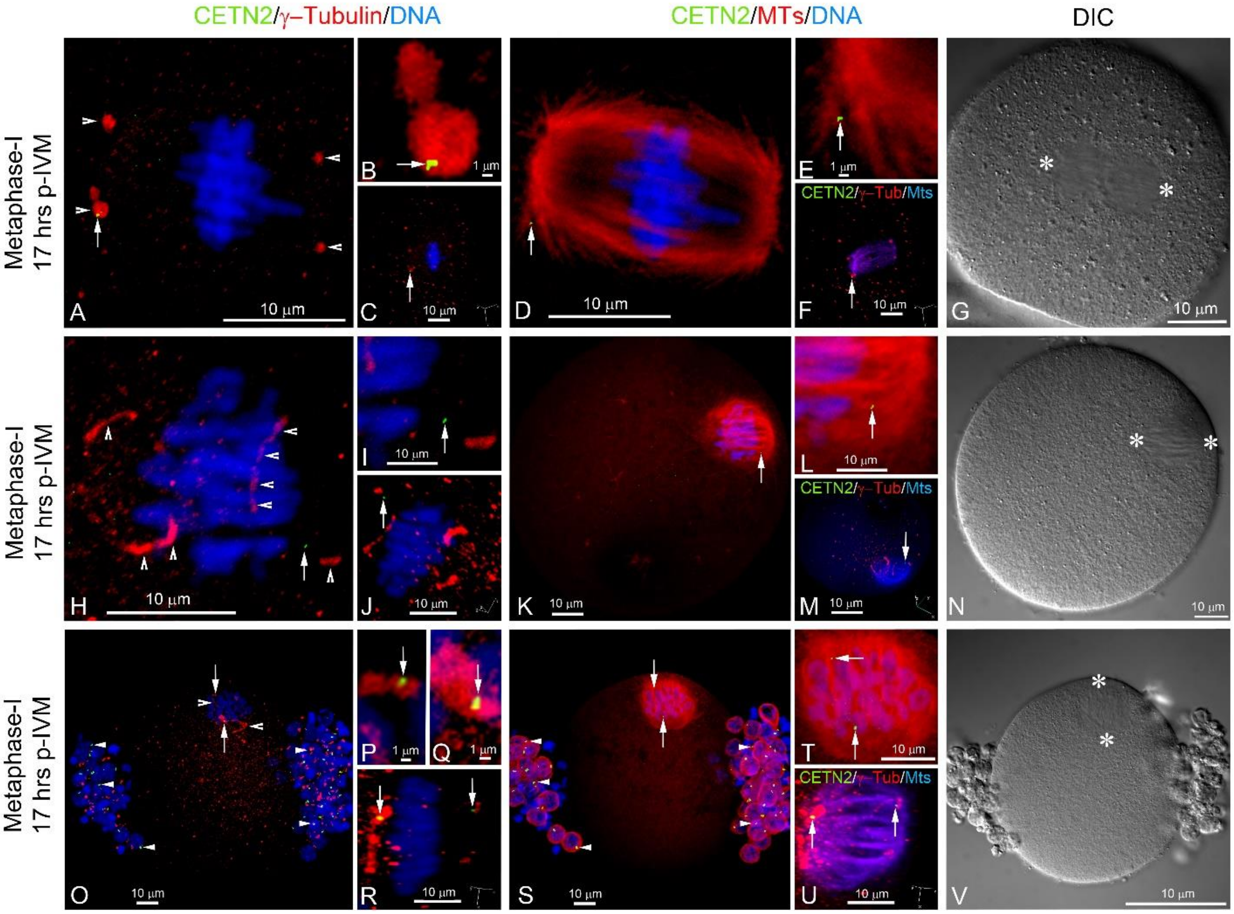

Supplemental Figure S3. Characteristics of GFP-tagged CETN2 centrosomes during oocyte growth and meiotic maturation. (**A**): Percentage of oocytes with GFP CETN2-expressing centrosomes, defined as GFP CETN2 foci embedded in γ-tubulin, at the indicated developmental stages. GFP CETN2 centrosomes were present in all incompetent GVs released from follicles (left two bars), but slightly reduced in competent mature GV oocytes (third bar). Meiotic maturation significantly reduced GFP CETN2-expressing centrosomes (right three bars) with only ~39% of metaphase-II arrested oocytes having detectable GFP CETN2 expression. Bars: mean ± standard deviation for minimum of 2 trials. N= number of oocytes scored. (**B**): Estimation of GFP CETN2 centriole areas (in μm^2) during oocyte growth to full competency. GFP CETN2 centriolar areas remain stable through oocyte development to full competency (bars, green). N= number of measured GFP CETN2 foci. (**C**): GFP CETN2-expressing centrosome localization during oocyte growth showing GV residing GFP CETN2 centrosomes in pre-antral follicle GVs (left bar, blue) but a transition to the cytoplasm (black) or cortical (gray) regions during follicular growth (middle bar, black). GFP CETN2 –expressing centrosomes return to the GV nucleus in mature competent GV oocytes (right bar, blue) prior to meiosis

resumption. N= number of oocytes. (**D**): Compared with arrested mature GVs (left bar, red), γ–tubulin PCM (D: red bars) surrounding GFP CETN2 expressing centrioles increases in area (in μm^2) following resumption of meiotic maturation, especially at metaphase-I. N= number of measured γ–tubulin foci with GFP CETN2-expressing centrioles. (**E**) Unlike γ–tubulin, GFP CETN2 expressing centrioles significantly decrease in area during meiotic maturation to metaphase-II arrest (E: green bars). (**F**): distribution of GFP CETN2-expressing centrosomes is overwhelmingly found with prometaphase-I (left bar) and metaphase-I (middle bar) meiotic spindles (blue) and rarely in the cytoplasm (black) or cortex (grey) during maturation. At metaphase-II arrest, ~ 20% of the oocytes demonstrate GFP CETN2 expressing centrosomes split between the meiotic spindle poles (right bar, dark green) and the extruded first polar bodies (right bar, light green). N= number of oocytes. All p-values determined by the two-tailed Student's t-test (GraphPad Software, Inc; keys: upper right in graphs).

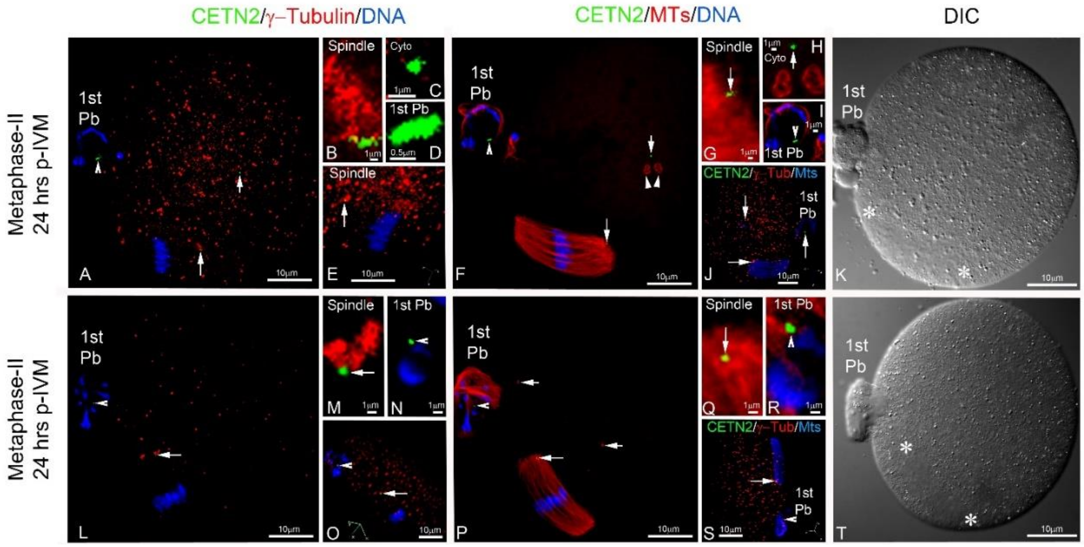

Supplemental Figure S4. GFP CETN2 expressing centrioles in metaphase-I meiotic spindles. (**A-G**): a bipolar metaphase-I spindle with two foci of γ–tubulin at each pole (A: red, arrowheads) but only one PCM foci at a single pole has a GFP CETN2 expressing centriole pair (A: green, arrow; B: details). The GFP CETN2 expressing centriole is localized to the extreme spindle pole edge (D: green, arrow; red, microtubules; E: details). C, F: rotational views (axis, lower right) for GFP CETN2 (green), γ–tubulin (red), and either DNA (C: blue) or microtubules (F: blue). (**H-N**): bipolar metaphase-I spindle with stretched ribbons of γ–tubulin at each spindle pole (H: red, arrowheads). The GFP CETN2 expressing centriole pair (H: green, arrow; I: details) is not associated with the γ–tubulin ribbons (red, arrowheads) but lies within the spindle lattice (K: arrow; red, microtubules; L: details) near the aligning bivalents (K: blue). J, M: rotational views (axis, lower right) for GFP CETN2 centriole pair (green), γ–tubulin (red), and either DNA (J: blue) or microtubules (M: blue). (**O-V**): a bipolar metaphase-I spindle with stretched ribbons of γ–tubulin at each spindle pole (O: red, arrowheads) with embedded GFP CETN2 centriole pairs (O: green, arrows; P, Q: details) located on the outside edges of the spindle lattice (S: arrows; red, microtubules: T: details). R, U: rotational views of GFP CETN2 (green), γ–tubulin (red), and either DNA (R: blue) or microtubules (U: blue). Adhering cumulus cells at the oocyte surface (solid arrowheads) also have co-localized GFP CETN2 expressing centrioles (green) embedded in γ–tubulin (red) and cortical microtubules (S: red; DNA, blue). All images are direct expressing GFP CETN2 oocytes counterstained for γ–tubulin (red), microtubules (cy5; color coded either red or blue) and DNA (blue). G, N, V: DIC, differential interference contrast with * denoting the first meiotic spindles. Bars, μm.

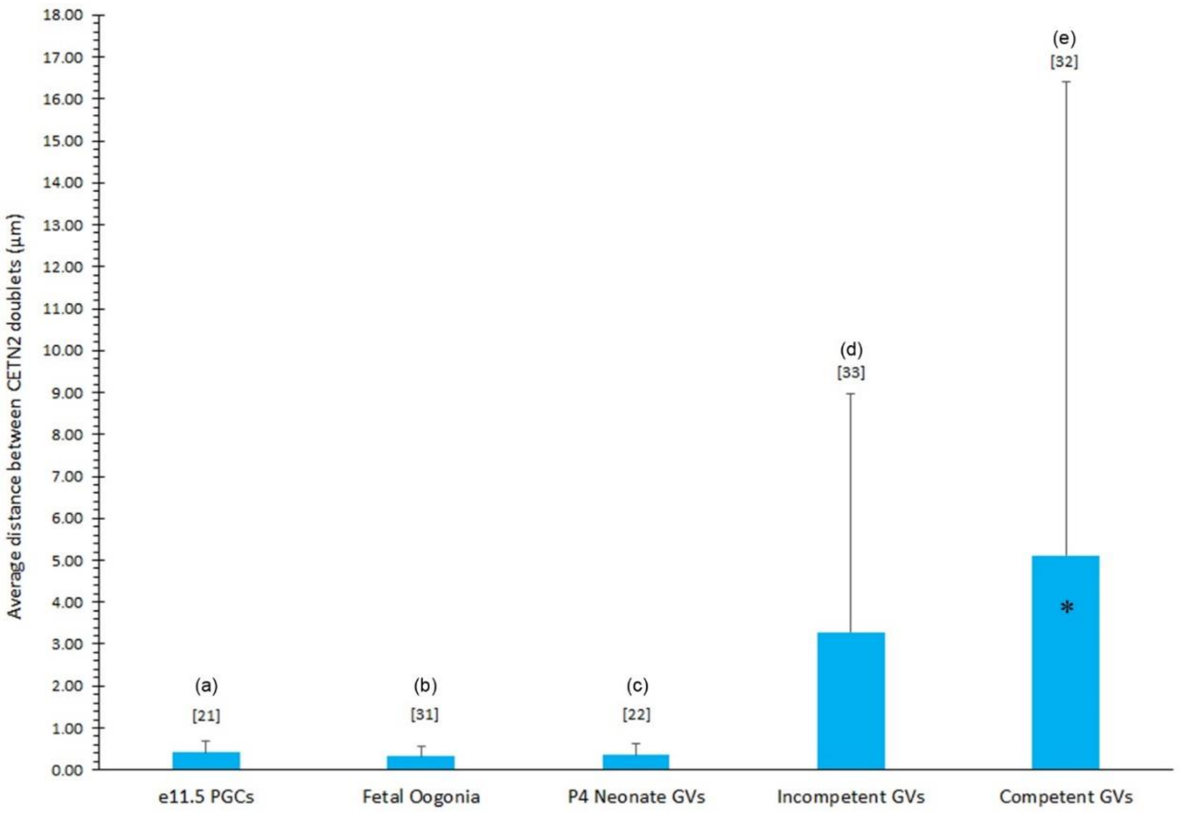

Supplemental Figure S5. Tracking the separation of GFP CETN2 doublet pairs in PGCs, oogonia, and GVs during follicular growth to full maturation. Analysis of distances (in µm) between GFP CETN2 expressing doublets in primordial germ cell, fetal oogonia and P4 neonate oocytes shows close alignment of the duplicated centrioles at G2 arrest (Fig. S5a-c, blue). With recruitment of follicular and oocyte growth at sexual maturity, GFP CETN2 doublets begin to separate within the pericentriolar material (PCM) and centriole doublet separation dramatically increases compared to P4 neonatal GFP CETN2 doublets (Fig. S5d, blue). This pattern is maintained until the oocyte reaches full maturity to resume meiotic resumption (Fig. S5e, blue; *: $p< 0.05$ compared to P4 oocytes). The scale bars in Fig. S5d-e reflects the tremendous range in distances recorded between GFP CETN2 doublets in both incompetent and mature GVs during oocyte growth (range: 0.0 - 55.16 µm). Brackets indicate the total number of oocytes measured for GFP CETN2 distance within each oocyte class with a minimum of three trials performed. Incompetent GV's include primary oocytes observed from adult ovary sections as well as small and medium follicle GVs (<61 µm). Competent GV's include large follicle GVs and fully mature GVs (>68 µm).

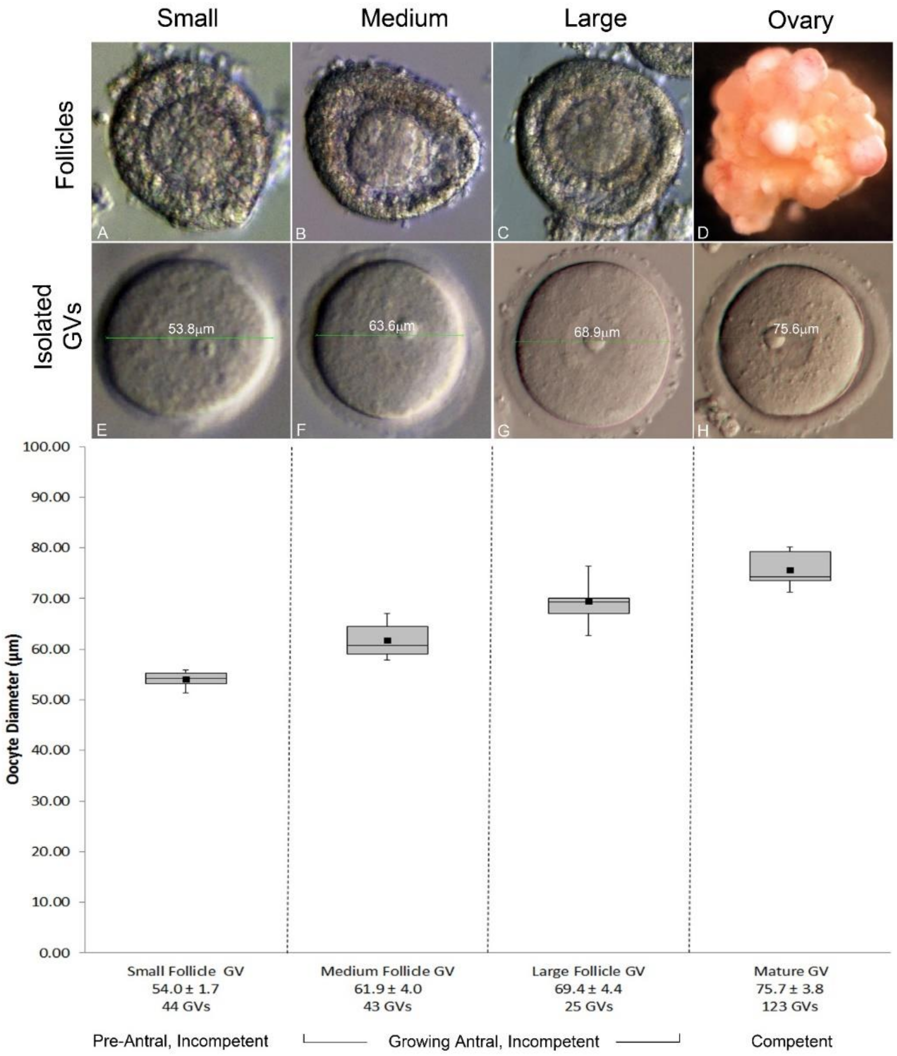

Supplemental Figure S6. Classification of Different Germinal Vesicle Stage Oocytes Harvested from GFP CETN2-Expressing CB6F1 Adult Ovaries. Upper panels show collection of follicle-enclosed oocytes harvested from adult ovaries and separated into small preantral (A), medium (B) or large antral (C) and fully mature antral follicles (D). Bottom panels: typical oocytes released from each follicle class and their respective diameters in μm, including small, incompetent oocytes (E); medium (F) and large (G) growing but incompetent oocytes; and fully mature competent GV oocytes (H). Graph: whisker plot data for diameter sizes of oocytes from each follicle class showing the median (horizontal line), upper 75th and lower 25th percentiles, means (black square), and maximum/minimum whiskers (bars). The means ± standard deviations and the total number of oocytes measured are provided below each whisker plot.

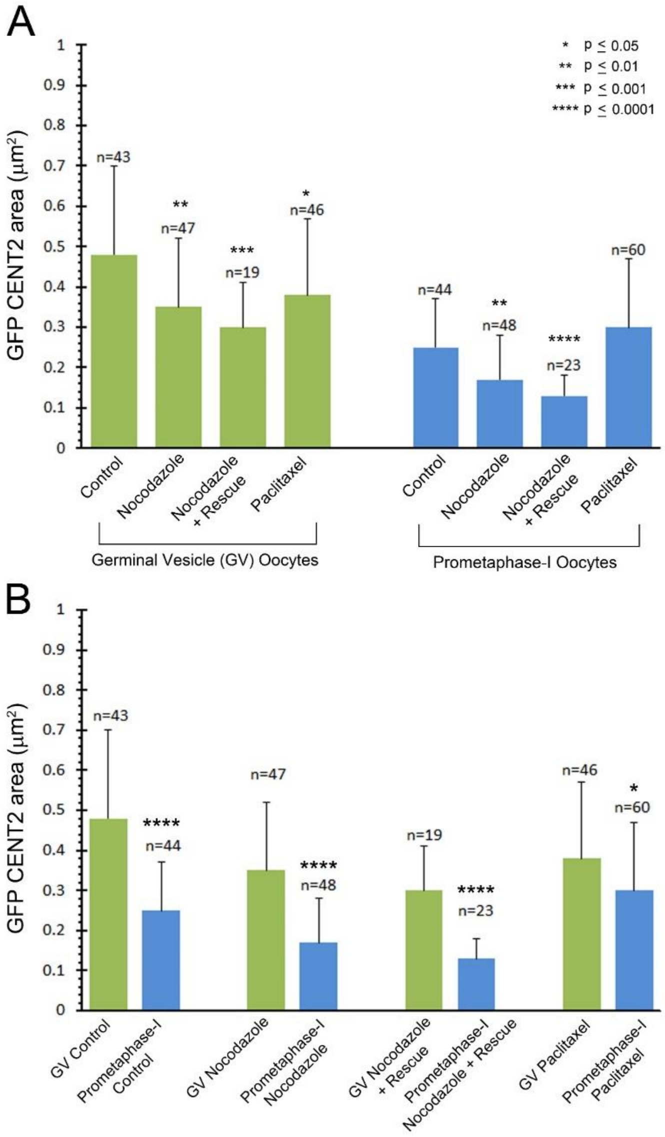

Supplemental Figure S7. Microtubule inhibitors alter GFP CETN2 foci areas in GV arrested oocytes and during meiotic maturation. GV oocytes, arrested from meiotic maturation resumption in 100 µg/ml dbcAMP, were culture in either 10µM nocodazole for 3.5 hr or 1µM paclitaxel for 15 min to disrupt microtubules. Rescue from nocodazole was performed by rinsing away the inhibitor and culturing for 5 min before fixation. Meiosis resumption was initiated by removing dbcAMP from the culture media and either developing in the continued presence of 10µM nocodazole for 3.5 hr or culturing for 3-hr:15min in medium before treating with 1µM paclitaxel in the final 15 mins prior to fixation. (**A**) Left bars: comparing GFP CETN2 foci areas in GV oocytes following exposure to nocodazole (second bar, green), recovery from nocodazole (third bar) or paclitaxel microtubule enhancement (fourth bar, green) shows

significant reduction in GFP CETN2 foci areas in all treatments compared to control GV's (first bar, green). Right bars: prometaphase-I produced in the continuous presence of nocodazole (second bar, blue) or recovered from nocodazole for 5 min at the end of inhibitor treatment (third bar, blue) showed significant reduction in GFP CETN2 foci areas. However, paclitaxel for 3.5hrs (fourth bar, blue) did not significantly alter GFP CETN2 foci areas analyzed against prometaphase-I control oocytes (first bar, blue). (**B**) the above data in A comparing control or microtubule inhibitor exposure in arrested GVs against prometaphase-I oocytes. GFP CETN2 foci areas significantly decrease after GVBD in control (first bars), nocodazole (second bars), recovery from nocodazole (third bars) or paclitaxel (fourth bars) exposure at prometaphase-I, suggesting GFP CETN2 foci are unstable in either GV arrested or early maturing oocytes. n= number of GFP CETN2 foci areas analyzed. *: significant difference to untreated GV or prometaphase-I control oocytes as determined by Student's t-test. Significance scale is present in upper right. A minimum of 2 trials were performed for each drug treatment.

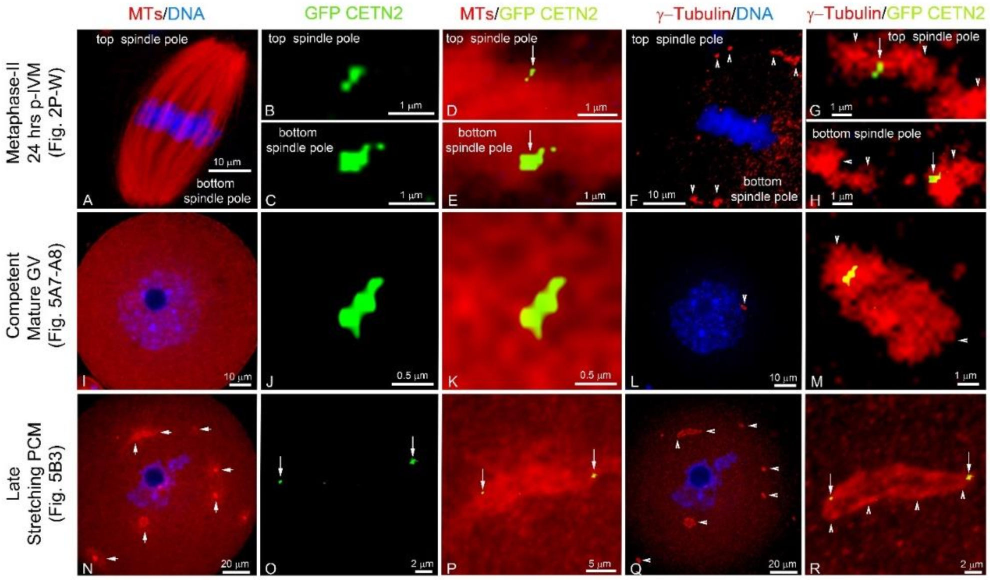

Supplemental Figure S8. Enhanced images of GFP CETN2 foci in associate with metaphase-II spindle poles and GV oocytes during growth. (A-H) z-projections in the metaphase-II-arrested meiotic spindle from Figure 2P-W. GFP CETN2 foci (B, C: green) are identified at opposite poles of the bipolar spindles (A: red, microtubules), with aligned chromosomes (A: blue) and γ-tubulin ribbons that encircle each pole (F: red, arrowheads). D, E: overlays of GFP CETN2 (green, arrows) with upper and lower spindle pole microtubules (red, microtubules). G, H: overlays of GFP CETN2 foci (green, arrows) with upper and lower spindle pole γ-tubulin ribbons (red, arrowheads). (**I-M**) Selected confocal z-projections of the mature competent GV oocyte shown in Figure 5A7-A8. GFP CETN2 centrioles (J: green) and the γ-tubulin foci (L: red, arrowhead) lying at the GV nucleus (I, L: blue, DNA) within sparse cortical microtubules (I: red). K: overlay of GFP CETN2 (green) with cytoplasmic microtubules (red). M: overlay of GFP CETN2 (green) with the slightly expanded γ-tubulin foci (red, arrowheads). (**N-R**): z-projection of the GV-residing MTOC expansion on the nuclear surface prior to meiosis resumption as shown in Figure 5B3. Microtubules (N: red, short arrows) assemble from γ-tubulin foci (Q: red, arrowheads). GFP CETN2 foci (O: green, arrows) associate with the largest MTOC, with the doublets splitting to opposite ends of the elongating MTOC (R: green, arrows; red, γ-tubulin, arrowheads). Other MTOCs on the GV nuclear surface lacking GFP CETN2 foci have not expanded (Q: red, arrowheads). P: overlay of split GFP CETN2 doublets (green, arrows) within the assembling microtubule aster (red). All images are of directly expressing GFP CETN2 oocytes (green), counterstained for γ-tubulin (red), microtubules (cy5, color assigned red), and DNA (blue). Bars in μm.

SUPPLEMENTAL MOVIES.

Supplemental Movie S1: Oocyte maturation. Oocyte overexpressing mCherry-Plk4 (magenta) and GFP-CETN2 (green) extruding a first polar body. Time steps= 1h. In this example, the GFP-CENT2 dots are retained within the polar body.

Supplemental Movie S2: Oocyte overexpressing mCherry-Plk4 (magenta) and GFP-CETN2 (grey) observed from NEBD to metaphase I. Time steps= 1h. Arrows point to the CETN2 foci.

Supplemental Movie S3: Oocyte overexpressing mCherry-Plk4 (magenta) and GFP-CETN2 (grey) observed from NEBD to spindle bipolarization. Time steps= 1h. Arrows point to the CETN2 foci.
